# Case report: tolvaptan-associated creatine kinase elevation in two patients with autosomal dominant polycystic kidney disease (ADPKD)

**DOI:** 10.1007/s00228-020-02906-z

**Published:** 2020-06-08

**Authors:** I. Agraz-Pamplona, M. Larrosa-Garcia, R. P. Bury-Macias, D. Serón-Micas, J. B. Montoro-Ronsano

**Affiliations:** 1 Division of Nephrology, Vall d’Hebron University Hospital, Barcelona, Spain; 2 Hospital Pharmacy, Vall d’Hebron Hospital Campus, Barcelona, Spain

Autosomal dominant polycystic kidney disease (ADPKD) is a genetic disorder that leads to an abnormal polycystin protein, which causes hypertension and cysts in different organs. The goal in ADPKD is to postpone kidney damage; however, there are no specific treatments [1].

Vasopressin antagonists decreased cAMP and cell proliferation in kidney epithelial cells and improved renal function in ADPKD-rodent models [2]. Tolvaptan (Jinarc®, Otsuka Pharmaceutical, Japan), an oral vasopressin-antagonist used for SIADH, slowed cyst development in ADPKD patients during TEMPO 3:4 and 4:4 clinical trials; adverse events (AE) were polydipsia, headache, polyuria, and fatigue [3–5]. It was approved for ADPKD in 2015 [6]; it is recommended to start treatment with 45 mg in the morning and 15 mg in the evening (45-0-15) and increase it according to tolerance.

## Case 1

A 41-year-old man was diagnosed with familial ADPKD at the age of 34. He had cysts in kidney and liver, and showed no symptoms other than hypertension. His kidney function was acceptable, 1.2 mg/dL mean creatinine plasma concentration (Cr). On 1 December 2018, his Cr was 1.66 mg/dL, and there was an increase in number and size of the cysts; due to the rapidly progressive ADPKD (clearance decrease > 5 mL/min/1.73m^2^/year) tolvaptan was started. The 45-0-15 dose of tolvaptan was well-tolerated, and the patient only reported thirst (an expected AE). After 20 days of treatment, the recommended blood test to evaluate tolvaptan-associated hepatotoxicity [6] showed a dramatic increase of creatine kinase (CK) plasma concentration from 256 UI/L (before tolvaptan) to 585 UI/L (normal range 22–198 UI/L). Lactate dehydrogenase (LDH) concentration was 432 UI/L (normal values 208–387UI/L), Cr did not vary, and the patient did not show clinical symptoms. The nephrologist did not find a cause for CK elevation, so it was decided to stop tolvaptan. Four weeks after, CK and LDH plasma levels recovered to basal level, and the patient reported feeling better than before.

## Case 2

A 43-year-old man was diagnosed with familial ADPKD at the age of 34. His kidneys were bigger than normal and contained numerous cysts; he had no liver cysts and normal laboratory tests with slightly high Cr (1.1 mg/dL). Disease symptoms were controlled for 5 years with enalapril, amlodipine, and allopurinol. In December 2018, infinite bleeding renal cysts were detected, and Cr was 1.55 mg/dL. Due to the ADPKD rapid progression, tolvaptan was initiated on 4 May 2019 at the regular dose, 45-0-15. After 17 doses of treatment, blood tests revealed a 5–6 fold increase of CK (from 154 to 854 UI/mL) and a moderate increase in LDH (364 UI/L). Cr did not increase during this time, and the patient did not have symptoms. Tolvaptan was stopped, while other treatments continued, and CK and LDH (307UI/L) gradually recovered after 6 weeks.

**Fig. 1:**
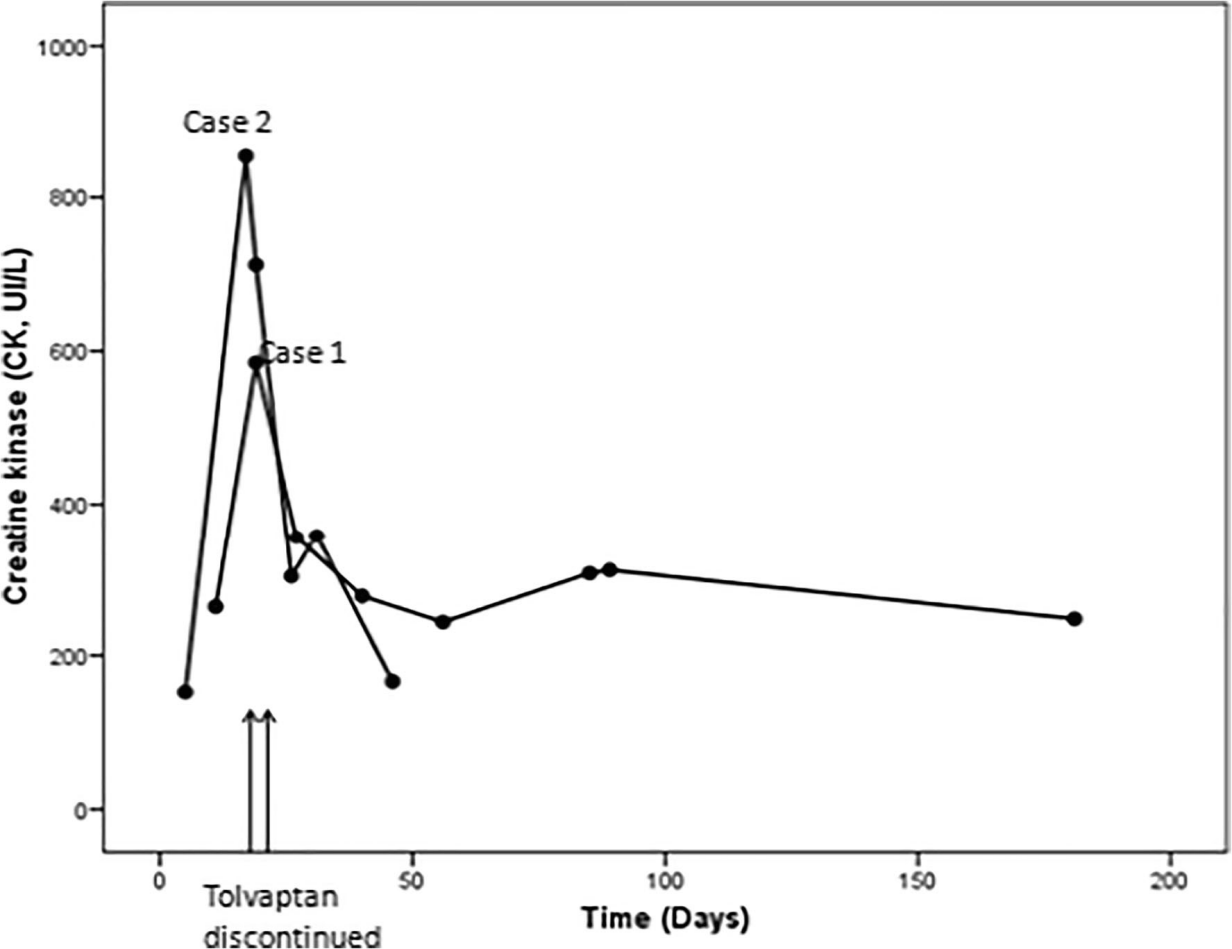
Creatine kinase (CK) plasma concentration during and after tolvaptan treatment. Case 1 and Case 2 are shown. Lines indicate day of treatment discontinuation

Both patients suffered a dramatic CK elevation after receiving tolvaptan and neither showed symptoms. They had no history of CK elevation and reported not having taken any other drug, phytotherapy, or special diet and not having performed extreme exercise that could justify CK elevation [7]. The LDH increase was minor; however, it is usually high in ADPKD due to cyst breakage. In the absence of any other justification for CK elevation and since CK decreased after tolvaptan discontinuation, it is probable that tolvaptan caused CK elevation in both cases [8] (Fig. 1).

These are the first cases of tolvaptan-induced CK elevation reported. It is well-known that tolvaptan causes hepatotoxicity in ADPKD patients [9] but not in other patients [10]. Since CK elevation is caused by muscle damage [7], we hypothesize that tolvaptan might damage ADPKD patients’ myocytes and hepatocytes by the same mechanism, probably related with intracellular cAMP reduction. Since CK elevation might be underestimated in ADPKD patients receiving tolvaptan, we suggest that CK should be monitored in these patients.
